# The Existence of a Sticking Region in Free Weight Squats

**DOI:** 10.2478/hukin-2014-0061

**Published:** 2014-10-10

**Authors:** Roland van den Tillaar, Vidar Andersen, Atle Hole Saeterbakken

**Affiliations:** 1 Department of Teacher Education of Nord Trøndelag University College, Levanger Norway.; 2 Department of Teacher Education and Sports of Sogn and Fjordane University College, Norway.

**Keywords:** EMG, muscle activity, kinematics, strength

## Abstract

The aim of this study was to investigate the existence of the sticking region in two legged free weight squats. Fifteen resistance-training males (age 24 ± 4 years, body mass 82 ± 11 kg, body height 179 ± 6 cm) with 6 ± 3 years of resistance-training experience performed 6-RM in free weight squats. The last repetition was analyzed for the existence of a sticking region. Only in 10 out of 15 participants a sticking region was observed. The observed sticking region was much shorter than in the bench press. Furthermore, rectus femoris decreased the EMG activity in contrast to increased EMG activity in biceps femoris around the sticking and surrounding region. No significant change in EMG activity was found for the lateral and medial vastus muscles. It is suggested that a combination of these muscle activity changes could be one of the causes of the existence of the sticking region in free weight squats.

## Introduction

**Figure 1:**
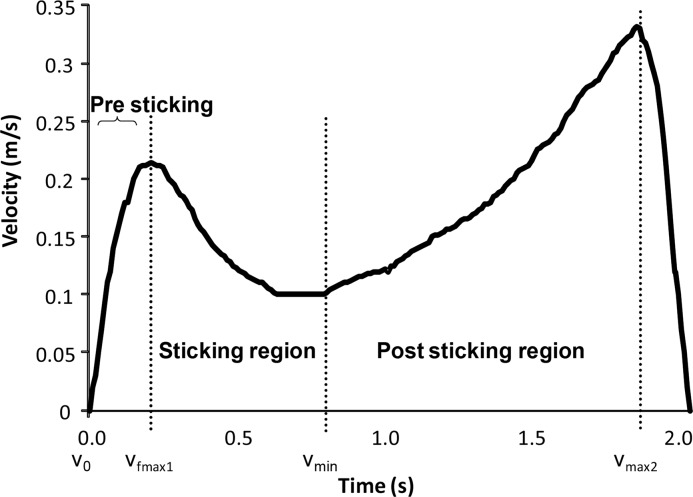
Typical vertical barbell velocity at maximal or near maximal attempts in the bench press with a pre sticking, sticking and post sticking region and following events: lowest position barbell (v_0_), first maximal barbell velocity (v_max1_), first located lowest barbell velocity (v_min_) and second maximal barbell peak velocity (v_max2_)

In strength training, squatting is one of the most used exercises for the lower body. Typically, the barbell is lowered to desired depth (i.e. quarter – half – or full squat) and moved upwards to fully extended knees (Sandler, 2005). A successful performance in squat is measured if the barbell is first lowered and then moved upwards again to extended position. However, when the weight cannot be moved all the way upward, it is considered a failed repetition. In several strength training exercises (i.e. bench press, deadlift, dumbbell chest press) this often occurs in a sticking region (Lander et al., 1985; Elliott et al., 1989; Newton et al., 1997; Escamilla et al., 2000; van den Tillaar and Sæterbakken, 2012). The sticking region is referred to as the region from the initial maximal upwards velocity (vmax1) to the first local minimum of the upward velocity (vmin) of the barbell (Madsen and McLaughlin, 1984). After this region the velocity increases again (Figure 1). In the bench press the sticking region only occurs during the upward (concentric) phase of the movement at maximal and near-maximal loads (Lander et al., 1985; Elliott et al., 1989; van den Tillaar and Ettema, 2009; 2010) and when fatigued (Duffey and Challis, 2007; van den Tillaar and Ettema, 2013).

Little is known of what causes this sticking region in resistance exercises. In the bench press, Elliott et al. (1989), Madsen and McLaughlin (1984), van den Tillaar et al. (2012) and van den Tillaar and Ettema (2013) have suggested that the sticking region is a poor mechanical force position in which the lengths and mechanical advantages of the muscles involved reduced the capacity to exert force in this region.

In the bench press the muscle activities were measured in the pre sticking region and post sticking to investigate if particular muscles were responsible of getting the participants surpass the sticking region (van den Tillaar and Ettema, 2010; van den Tillaar et al., 2012; van den Tillaar and Ettema, 2013). When fatigue occurred in the 6-RM bench press, the triceps brachii had similar muscle activity during these regions which demonstrated that the muscle was not responsible for getting the participants out of the sticking region (van den Tillaar and Sæterbakken, 2013). This was conferred by other studies in the bench press with maximal load (van den Tillaar et al., 2012; van den Tillaar and Ettema, 2013) and shown that the deltoid and pectoralis major muscles were responsible, and not the triceps brachii, for getting the lifter out of the sticking region in the bench press.

To our best knowledge, no studies have investigated occurring of a sticking region in squatting. Investigating the kinematics and muscle activation during the presumed sticking region of free weight squats would provide information about possible causes of the sticking region in squats. Further, it can help understand which muscles may be responsible to get the barbell through the sticking region.

Therefore, the aim of this study was to investigate the existence of the sticking region in two legged free weight squats. We investigated the kinematics and muscle activity in the ascending part around the sticking region during the last repetition during 6-RM squats in men with recreational resistance training backgrounds. It was hypothesized that the sticking region occurred and that the muscle activity of the prime movers was lower in the sticking region than preand post sticking regions.

## Material and Methods

### Participants

The study sample consisted of fifteen healthy males (age 24 ± 4 years, body mass 82 ± 11 kg, body height 179 ± 6 cm) with 6 ± 3 years of resistance-training experience who performed 6-RM (135 ± 33kg) in free weight squats. None of them was a competitive power- or weight lifter. Inclusion criteria were being able to lift 1.5 of their own bodyweight in squat (femur parallel to the floor) with good full squatting technique and no injuries or pain which could reduce their maximal performance. The participants did not follow any resistance training of the legs 72 hours before testing. All the subjects were informed verbally and in writing of the procedures and possible risks of the tests and provided written consent before they were included in the study. The study complied with the requirements of the regional Committee for Medical Research Ethics and conformed to the latest revision of the Declaration of Helsinki.

### Procedures

To investigate the kinematics and muscles patterning during the sticking region in squats the last repetition of 6-RM free weight squats was analyzed. 6-RM was used since this is a typical training load to increase maximal strength (ASCM position stand, 2009). In addition, it is also safer to conduct 6-RM than maximal 1-RM lifts in squats, 1-RM are rarely used in training and van den Tillaar and Saeterbakken (2012) showed that in the 6-RM bench press a typical sticking region occurs during the last repetition during 6-RM lifts.

Two familiarization tests were conducted two weeks before the experimental test. In the first familiarization test, the subject placed their feet in their preferred position (to avoid extra stress upon the subject and increase the external validity towards training) in which the position of the feet was measured. This position was then controlled and identical in every later session. Then the lower position (defined as 80 degrees in the knee joint, full extension defined as 180 degrees) was found using a protractor. A horizontal rubber band was used to identify this lower position during the tests which the participants had to touch with their proximal part of hamstring before starting the upwards movement.

In the first familiarization test, the 6-RM load was estimated by the participants. The subjects reported their estimated 6-RM and 95% of this load was used. In the second familiarization session and the experimental test, testing started at the 6-RM achieved in the first familiarization session. The load was increased or decreased by 2.5 kg or 5 kg until the real 6-RM was obtained (1–3 attempts). Between three to five minutes rest was given between each attempt (Goodman et al., 2008). Before testing and after a general warm-up on a treadmill or cycle, the participants performed a progressive specific warm-up protocol according to the same protocol as described by Paoli et al. (2009) and Saeterbakken and Fimland (2013a). It consisted of 15 repetitions at 30%, 10 repetitions at 50% and 6 repetitions at 80% of 6-RM in squatting.

The 6-RM squats were performed in a power rack (Gym 2000, Modum, Norway) with an Olympic barbell (2.8 cm diameter, length 1.92 m). The participants bended from full knee extension in a self-paced, but controlled tempo until the back of their thigh touched the rubber band. They then received a verbal signal from the test-leader and returned to the starting position.

### Measurements

A linear encoder (ET-Enc-02, Ergotest Technology AS, Langesund, Norway) connected to the barbell measured the lifting time of the barbell with a resolution of 0.075 mm and counted the pulses with 10 millisecond intervals (Arnason et al., 2004). The vertical displacement was measured in relation to the lowest point of the barbell (zero distance). Velocity of the barbell was calculated by using a five point differential filter with software Musclelab V8.13 (Ergotest Technology AS, Langesund, Norway). A goniometer (Biometrics SG150, Biometrics Ltd, Newport, UK) was attached and aligned to the knee joint to measure the knee angle during the squat. The knee angle, barbell displacement and velocity were identified at the following positions in the upwards movement of the squat: lowest position of the barbell (v_0_), first maximal barbell velocity (v_max1_), first located lowest barbell velocity (v_min_) and second maximal barbell peak velocity (v_max2_) (Figure 1). The linear encoder was synchronized with the goniometer and EMG recordings using a Musclelab 4020e and analyzed by software V8.13 (Ergotest Technology AS, Langesund, Norway).

EMG activity of the vastus lateralis, vastus medialis, rectus femoris, biceps femoris and soleus muscles was measured. Before placing the gel coated self-adhesive electrodes (Dri-Stick Silver circular sEMG Electrodes AE-131, NeuroDyne Medical, USA), the skin was shaved, abraded and washed with alcohol. The electrodes (11 mm contact diameter and a 2 cm center-to-center distance) were placed along the presumed direction of the underlying muscle fiber according to the recommendations by SENIAM or similar studies (Hermens et al., 2000; Saeterbakken and Fimland, 2012; 2013a). The electrodes were placed on the dominant leg (Saeterbakken and Fimland, 2013b). To minimize noise from the surroundings, the raw EMG signal was amplified and filtered using a preamplifier located close to the sampling point. The preamplifier had a common mode rejection ratio of 100 dB, high cut frequency at the level of 600 Hz and low cut frequency at the level of 8 Hz. The EMG signals were converted to root mean square (RMS) EMG signals using a hardware circuit network (frequency response 0 – 600 kHz, averaging constant 100 ms, total error ± 0.5%). Finally, the RMS converted signal was sampled at 100 Hz using a 16 bit A/D converter. Commercial software (MuscleLab V8.13, Ergotest Technology AS, Langesund, Norway) was used to analyze the stored EMG data.

In order to compare muscle activity during the upward bench press movement, three regions were assigned. The first region (pre sticking region was from the lowest barbell point (v_0_) until the maximal barbell velocity (v_max1_); the next region (sticking region) was from the maximal barbell velocity until the first located lowest vertical barbell velocity, also called the sticking point (v_min_); the last region, the post-sticking region, started at first located lowest barbell velocity to the second maximal barbell peak velocity (v_max2_), which was also called the strength region (Figure 1) (Lander et al., 1985; van den Tillaar and Saeterbakken, 2012). Only the root mean square (RMS) EMG of each region in each subject that showed a sticking region in their last repetition was calculated and used for further analysis.

### Statistical analysis

A one-way analysis of variance (ANOVA) with repeated measures was used with Holm-Bonferroni post-hoc tests to assess differences in the EMG activity for each of the muscles. In case that the sphericity assumption was violated, the Greenhouse-Geisser adjustments of the p-values were reported. Statistical analyses were performed with SPSS version 21.0 (SPSS, Inc., Chicago, IL). All results are presented as means ± standard deviations. Statistical significance was accepted at *p*≤0.05.

## Results

**Figure 2:**
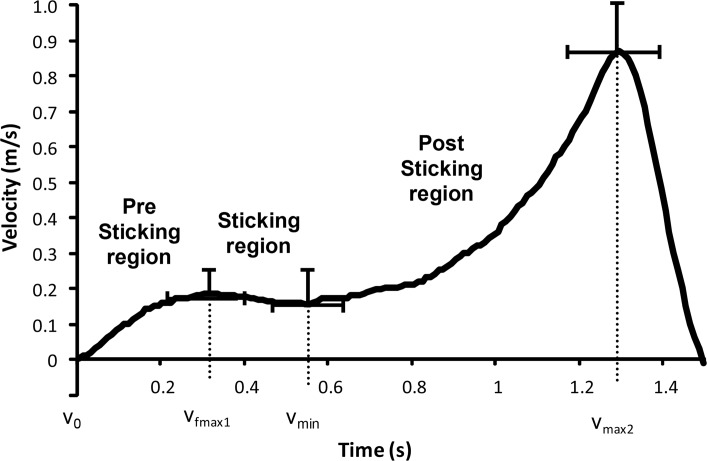
Typical vertical barbell velocity (± SD) in the last repetition in 6-RM squatting with a pre sticking, sticking and post sticking region and following events: lowest position barbell (v_0_), first maximal barbell velocity (v_max1_), first located lowest barbell velocity (v_min_) and second maximal barbell peak velocity (v_max2_)

**Figure 3:**
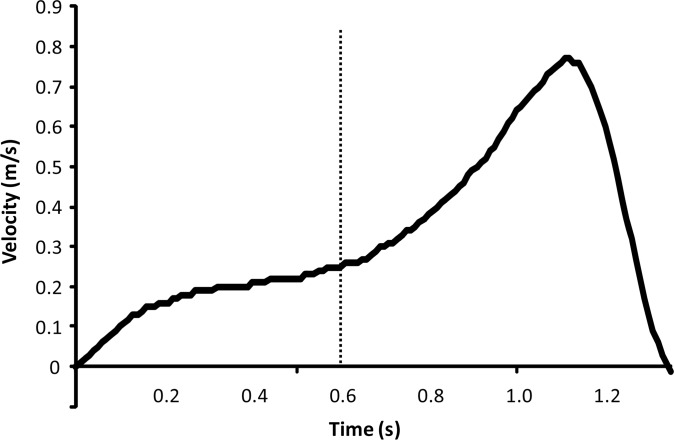
Typical vertical barbell velocity for participants that did not show a sticking region, therefore a velocity development that can be divided into two clear regions shown by the dashed line

The lifted 6-RM load was 137 ± 28 kg. However, only in ten of the fifteen participants a clear sticking region was observed during the last repetitions of the 6-RM lift (Figure 2). Figure 2 shows a typical example of the development of the velocity during the squat exercise with the sticking region from v_max1_ to v_min_. After the v_min_ the velocity increased again and all participants (that showed a sticking region) obtained a clearly higher peak velocity after the v_min_ than before the V_min_. The sticking region lasted for 0.21 ± 0.10 s and v_min_ occurred on average after around 0.54 ± 0.24 s at a height. The sticking region started at 0.10 m ± 0.04 from the deepest point of the barbell (Table 1). The velocity increased first to 0.174 m/s at v_max1_, afterwards, it decreased with only 0.032 m/s to 0.142 m/s at v_min_. Thereafter it increased rapidly again to a maximum of 0.723 m/s at v_max2_ (Figure 2). The knee joint angles were 89, 98, 102 and 136 degrees at v_0_, v_max1_, v_min_ and v_max2_, respectively (Table 1). The other five participants that did not show a clear sticking region had another barbell velocity development as may be seen in Figure 3.

**Figure 4:**
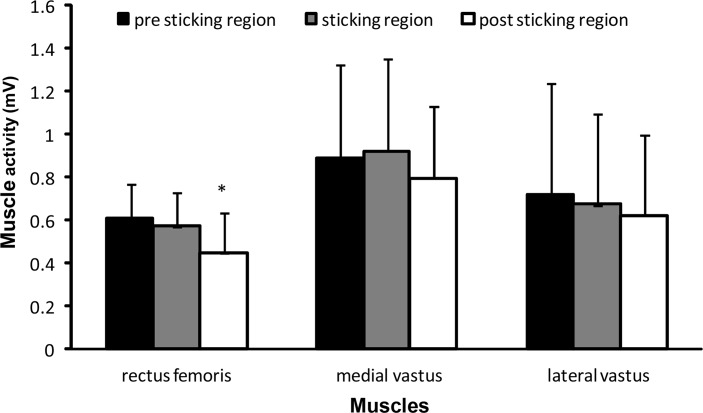
Mean (± SD) root mean square (RMS) EMG activity of pre sticking, sticking and post sticking region in rectus femoris, vastus lateralis and medialis during the last repetition of 6-RM squatting. * indicates significant difference with all other regions at p ≤ 0.05

**Figure 5:**
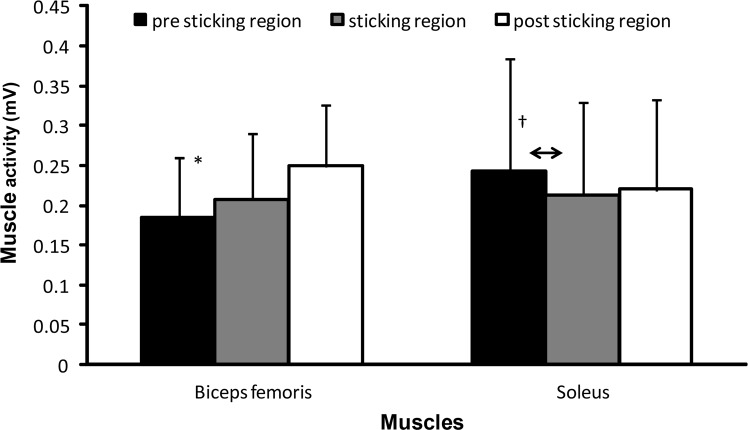
Mean (± SD) root mean square (RMS) EMG activity of the pre sticking, sticking and post sticking region in biceps femoris and soleus during the last repetition of 6-RM squatting. * indicates significant difference with all other regions at p ≤ 0.05. † indicates significant difference between these two regions at p ≤ 0.05

Only on the subjects that showed sticking regions a one-way ANOVA for repeated measures on EMG of the different muscles was performed. The results indicated significant main effects for the rectus femoris (F=10.45; p=0.001; Figure 4) and biceps femoris (F=5.75; p=0.012; Figure 5) muscle activity during the three regions. Post hoc comparisons revealed that for the rectus femoris the activity significantly increased from the sticking region to the post-sticking region (Figure 4). The biceps femoris muscles increased their activity significantly from the pre sticking to the other two regions (Figure 5). No significant effect was found for the soleus muscle (F=2.74; p=0.094; Figure 5). However, post hoc comparison showed that the soleus muscle activity significantly decreased from the pre sticking region to the sticking region (p=0.033; Figure 5). The other muscles did not significantly change their muscle activity during the three regions in the squat (F≤2.214; p≥0.138; Figures 4–5).

## Discussion

The purpose of the study was to examine the existence of a sticking region and concomitant neuromuscular activation during free weight squats. The main finding was that the majority of the participants exhibited a sticking region during the last repetition of 6-RM squatting, but not all. Furthermore, only the biceps femoris activity increased from the pre- to sticking region, while the rectus femoris increased in the post sticking region (Figures 4–5).

Only in ten of the fifteen participants a sticking region was observed during the last repetition of the 6-RM. It could be speculated that these participants had a very effective lifting technique in which they surpassed the sticking region. Figure 3 shows a typical example of a subject that did not show a sticking region. It may be seen that the velocity development could be divided into two phases: the first phase in which the velocity increased, but not rapidly (before the dashed line) and the second phase in which the velocity increased rapidly until reaching the maximal values. It could be speculated that first the knee extended, followed up by the hip extension that resulted in this velocity development. In the present study only the knee angle was measured and not the hip and ankle angles, what made it difficult to state if this was the case in the participants. Another explanation is that these five participants did not lift their actual 6-RM, but a lower percentage. For example Newton et al. (1997) found that there was no sticking region in lifts with a lower percentage than 85% of 1-RM lifts in the bench press. However, in the present study the participants had two familiarization sessions before the experimental test and they had one to three attempts to establish their real 6-RM. Thus, it would be unlikely that this was the case in our study.

To our best knowledge this is the first study that has evaluated the sticking region in squats, which makes it difficult to compare the present findings with other studies about this region. The development of velocity was similar to that observed in the bench press with first a low peak that occurred between 0.2 (counter movement) and 0.6 s (pure concentric) in the upwards part of the maximal lifts (van den Tillaar and Ettema, 2013). Then, the sticking region occurred, followed by a second higher peak velocity (van den Tillaar and Ettema, 2009). However, these two phases (sticking and post sticking region) were different between the bench press and squats. The sticking region was much shorter in time (0.2 vs. 0.8 s) and also the velocity decreased less (0.03 vs. 0.22 m/s) (van den Tillaar and Saeterbakken, 2012) (Figure 2). These differences may be explained by size of the muscle involved in the different lifts. In the bench press the involved muscle mass which is responsible for surpassing the sticking region is much smaller than the muscle mass in the squats. This makes it easier to generate more force if necessary (Bruche et al., 2002). When compared to deadlift kinematics, the sticking region is longer and the second peak velocity is lower than the first peak velocity (Escamilla et al., 2000; Hales et al., 2009), which was the opposite of the velocity development in the squats. The differences in these sticking regions are suggested to be the result of the type of lift. The squat represents a synergistic, simultaneous movement, whereas the deadlift consists of a sequential movement (Hales et al., 2009). Furthermore, squats include a downward and upward movement, while in the deadlift the lifter only performs an upward (concentric) movement. In the bench press it was found that this (counter vs. concentric movement) had an effect upon the sticking region (van den Tillaar and Ettema, 2013).

None of the measured muscles EMG activity was lower during the sticking region compared to the other regions (Figures 4–5), which was contrary to our hypothesis. Only the soleus muscle activity decreased from the pre sticking to sticking region (Figure 3). This can be explained by the fact the soleus function is plantar flexion in the ankle. During the first part of the lift this probably occurs, while later in the movement this would be inappropriate, because it would cause a movement backwards with the barbell. This would make it more difficult to lift the barbell straight up. The rectus femoris decreased muscle activity from the sticking to post sticking region, can also be explained by its function, which is to extend the knee and flex the hip joint. During the later part of the squat the hip is extending and, therefore, the activity of the rectus femoris has to be decreased in the post sticking region (Figure 4). For the biceps femoris the opposite happened; the biceps femoris has as function to flex the knee. Since the long head of the biceps femoris originates in the pelvis it is also involved in hip extension. For this reason the long head is a weaker hip extender when the knee is flexed due to active insufficiency (Marshall et al., 1972), which is the case during the pre-sticking region (Figure 5). Its activity increases when the knee is more extended during the sticking and post sticking region. It could be that the combination of the increased activity of the biceps femoris and the decreased activity of the rectus femoris is one of the causes of the existence of the sticking region.

In this study activity of the glutei muscles was not measured. These muscles are responsible for the hip extension during the lift. It could be that the glutei muscles are partly or mainly responsible for the existence of this sticking reason and that is perhaps why some exhibit this sticking region while it is not observed in others. As shown in the deadlift in which the glutei muscle is the most important muscle during the lift, also a sticking region was found (Escamilla et al., 2000; Hales et al., 2009), while in squats several muscle groups are important during the lift. Perhaps that is why we cannot explain existence of the sticking region with the current analyses of the measured muscle patterning. Furthermore, only the knee angle was measured, due to the availability of just one goniometer. This made it also difficult to investigate if the sticking region occurred due to the change from knee flexion to more hip extension or a combination of both which was not efficient in those subjects who showed a sticking region. Including several goniometers or 3D kinematics to measure the hip and ankle joint angle would give more detailed information about the lift and possible causes of the existence of the sticking region. Furthermore, by 3D kinematics measurements, moment arms can be calculated, which also could have an influence on the existence of the sticking region as suggested in the bench press (van den Tillaar and Ettema, 2013).

This is the first study in squats that investigated existence of the sticking region and muscle activation during the upwards part of the lift. However, due to the limitations of the study which stemmed from methodological issues and insufficient equipment, it is not possible to come with a conclusion about what the possible causes of the existence of the sticking region are. Some possible reasons like a combination of muscle patterning, inefficient moment arm and joint angles have been previously mentioned. Future studies should include more EMG measurements of other involved muscles (i.e. glutei muscles) and 3D measurements to establish more detailed information about these variables.

The gained information from the present study can help coaches, researchers and athletes in their understanding of the sticking region and limitation of the muscles during 6-RM free weight squats. Since it is not clear yet which muscles are responsible for surpassing the sticking region (the weakest region during the lift) and thereby would help in increasing free weight squat performance, it is not possible to come with a recommendation for specific training that could target specific muscles. More studies have to be conducted on this region in squats before coming with such a statement.

## Figures and Tables

**Table 1:** Mean variables with their standard deviation at lowest barbell point (v_0_), first maximal barbell velocity (v_max1_), minimal vertical barbell velocity (v_min_) and second peak barbell velocity (v_max2_) during the squat movement

**Variable**	**v_0_**	**v_max1_**	**v_min_**	**v_max2_**
Barbell velocity (m/s)	0	0.174±0.056	0.142±0.080	0.723±0.159
Barbell height (m)	0	0.067±0.035	0.103±0.043	0.371±0.046
Knee joint angle (º)	89±12	98±11	102±11	136±12
Time intervall (s)	0	0.380±0.108	0.216±0.100	0.707±0.154

## References

[b1-jhk-42-63] ACSM American College of Sports Medicine position stand (2009). Progression models in resistance training for healthy adults. Med Sci Sports Exerc.

[b2-jhk-42-63] Arnason A, Sigurdsson SB, Gudmundsson A, Holme I, Engebretsen L, Bahr R (2004). Risk Factors for Injuries in Football. Am J Sports Med.

[b3-jhk-42-63] Brechue WF, Abe T (2002). The role of FFM accumulation and skeletal muscle architecture in powerlifting performance. Eur J Appl Physiol.

[b4-jhk-42-63] Duffey MJ, Challis JH (2007). Fatigue effects on bar kinematics during the bench press. J Strength & Cond Res.

[b5-jhk-42-63] Elliott BC, Wilson GJ, Kerr GK (1989). A biomechanical analysis of the sticking region in the bench press. Med Sci Sports Exerc.

[b6-jhk-42-63] Escamilla RF, Fransisco AC, Fleisig GS, Barrentine SW, Welch CM, Kayes AV, Speer KP, Andrews JR (2000). A three-dimensional biomechanical analysis of sumo and conventional style deadlifts. Med Sci Sports Exerc.

[b7-jhk-42-63] Goodman CA, Pearce AJ, Nicholes CJ, Gatt BM, Fairweather IH (2008). No difference in 1RM strength and muscle activation during the barbell chest press on a stable and unstable surface. J Strength & Cond Res.

[b8-jhk-42-63] Hales ME, Johnson BF, Johnson JT (2009). Kinematic analysis of the powerlifting style squat and the conventional deadlift during competition: is there a cross-over effect between lifts?. J Strength & Cond Res.

[b9-jhk-42-63] Hermens HJ, Freriks B, Disselhorst-Klug C, Rau G (2000). Development of recommendations for SEMG sensors and sensor placement procedures. J Electr & Kin.

[b10-jhk-42-63] Lander JE, Bates BT, Swahill JA, Hamill J (1985). A comparison between free-weight and isokinetic bench pressing. Med Sci Sports Exerc.

[b11-jhk-42-63] Madsen N, McLaughlin T (1984). Kinematic factors influencing performance and injury risk in the bench press exercise. Med Sci Sports Exerc.

[b12-jhk-42-63] Marshall JL, Girgis FG, Zelko RR (1972). The biceps femoris tendon and its functional significance. J Bone Joint Surg Am.

[b13-jhk-42-63] Newton R, Murphy AJ, Humphries B, Wilson G, Kraemer W, Häkkinen K (1997). Influence of load and stretch shortening cycle on the kinematics, kinetics and muscle activation that occurs during explosive upper body movements. Eur J Appl Physiol.

[b14-jhk-42-63] Paoli A, Marcolin G, Petrone N (2009). The effect of stance width on the electromyographical activity of eight superficial thigh muscles during back squat with different bar loads. J Strength & Cond Res.

[b15-jhk-42-63] Saeterbakken AH, Fimland MS (2013a). Electromyographic activity and 6RM strength in bench press on stable and unstable surfaces. J Strength & Cond Res.

[b16-jhk-42-63] Saeterbakken AH, Fimland MS (2011). Muscle activity of the core during bilateral, unilateral, seated and standing resistance exercise. Eur J Appl Physiol.

[b17-jhk-42-63] Saeterbakken AH, Fimland MS (2013b). Muscle force output and electromyographic activity in squats with various unstable surfaces. J Strength & Cond Res.

[b18-jhk-42-63] Sandler D (2005). Sports Power.

[b19-jhk-42-63] van den Tillaar R, Ettema G (2009). A comparison of kinematics and muscle activity between successful and unsuccessful attempts in bench press. Med Sci Sports Exerc.

[b20-jhk-42-63] van den Tillaar R, Ettema G (2013). A comparison of muscle activity in concentric and counter movement maximum bench press. J Human Kin.

[b21-jhk-42-63] van den Tillaar R, Ettema G (2010). The “sticking period” in bench press. J Sports Sci.

[b22-jhk-42-63] van den Tillaar R, Sæterbakken A (2012). The sticking region in three chest-press exercises with increasing degrees of freedom. J Strength & Cond Res.

[b23-jhk-42-63] van den Tillaar R, Saeterbakken AH, Ettema G (2012). Is the occurrence of the sticking region the result of diminishing potentiation in bench press?. J Sports Sci.

[b24-jhk-42-63] van den Tillaar R, Sæterbakken AH (2013). Fatigue effects upon sticking region and electromyography in a six-repetition maximum bench press. J Sports Sci.

